# Vibration, acoustic, temperature, and motor current dataset of rotating machine under varying operating conditions for fault diagnosis

**DOI:** 10.1016/j.dib.2023.109049

**Published:** 2023-03-09

**Authors:** Wonho Jung, Seong-Hu Kim, Sung-Hyun Yun, Jaewoong Bae, Yong-Hwa Park

**Affiliations:** a Department of Mechanical Engineering, Center for Noise and Vibration Control Plus, Korea Advanced Institute of Science and Technology, 291, Daehak-ro, Daejeon, Yuseong-gu 34141, South Korea; b Automotive R&D Division, Hyundai Motor Group, 150, HyundaiYeonguso-ro, Namyang-eup, Hwaseong-si, Gyeonggi-do 18280, South Korea

**Keywords:** Ball Bearing, Unbalance, Misalignment, Load Fluctuation, Speed Fluctuation, Condition Monitoring

## Abstract

Rotating machines are often operated under various operating conditions. However, the characteristics of the data varies with their operating conditions. This article presents the time-series dataset, including vibration, acoustic, temperature, and driving current data of rotating machines under varying operating conditions. The dataset was acquired using four ceramic shear ICP based accelerometers, one microphone, two thermocouples, and three current transformer (CT) based on the international organization for standardization (ISO) standard. The conditions of the rotating machine consisted of normal, bearing faults (inner and outer races), shaft misalignment, and rotor unbalance with three different torque load conditions (0 Nm, 2 Nm, and 4 Nm). This article also reports the vibration and driving current dataset of a rolling element bearing under varying speed conditions (680 RPM to 2460 RPM). The established dataset can be used to verify newly developed state-of-the-art methods for fault diagnosis of rotating machines. Mendeley Data. DOI:10.17632/ztmf3m7h5x.6, DOI:10.17632/vxkj334rzv.7, DOI:10.17632/x3vhp8t6hg.7, DOI:10.17632/j8d8pfkvj2.7

**Specifications Table**

**Table:** 

Subject	Engineering – Mechanical Engineering
Specific subject area	Rotating Machine Condition Monitoring
Type of data	Time-series vibration data (x-axis, and y-axis) Time-series acoustic data Time-series temperature data Time-series driving current data (three-phases) Time-series speed data Tables Figures
How the data were acquired	Four ceramic shear ICP based accelerometers (PCB352C34) are mounted on x- and y-directions of two bearing housings based on ISO 10816-1:1995. An acoustic microphone (PCB378B02) is located nearby bearing housing based on ISO 8528-10. Two thermocouples (K-type) are mounted on bearing housings. Three CT sensors (Hioki CT6700) are installed on U-phase, V-phase and W-phase of main motor. Vibration and acoustic data are collected from the accelerometers and microphone by the SIEMENS SCADAS Mobile 5PM50. Temperature, and driving current data are collected from thermocouples and CT sensors by NI 9775 and NI 9211, respectively. The speed data were also collected from one tachometer (Autonics FD-620-10).
Data format	Raw
Description of data collection	This testbed can emulate bearing faults, shaft parallel misalignment faults and rotor unbalance faults. The dataset is composed of two parts. First dataset was acquired from the testbed under different load and constant rotating speed condition. Second dataset was acquired from the testbed under randomly varying rotating speed condition without load.
Data source location	· Institution: Human Lab., Center for Noise and Vibration Control Plus, Department of Mechanical Engineering, Korea Advanced Institute of Science and Technology (KAIST) · City: Daejeon · Country: South Korea
Data accessibility	Repository name: Vibration, Acoustic, Temperature, and Motor Current Dataset of Rotating Machine Under Varying Load Conditions for Fault Diagnosis Data identification number: 10.17632/ztmf3m7h5x.6 Direct URL to data: https://data.mendeley.com/datasets/ztmf3m7h5x Repository name: Vibration and Motor Current Dataset of Rolling Element Bearing Under Varying Speed Conditions for Fault Diagnosis: Subset1 Data identification number: 10.17632/vxkj334rzv.7 Direct URL to data: https://data.mendeley.com/datasets/vxkj334rzv Repository name: Vibration and Motor Current Dataset of Rolling Element Bearing Under Varying Speed Conditions for Fault Diagnosis: Subset2 Data identification number:10.17632/x3vhp8t6hg.7 Direct URL to data: https://data.mendeley.com/datasets/x3vhp8t6hg Repository name: Vibration and Motor Current Dataset of Rolling Element Bearing Under Varying Speed Conditions for Fault Diagnosis: Subset3 Data identification number: 10.17632/j8d8pfkvj2.7 Direct URL to data: https://data.mendeley.com/datasets/j8d8pfkvj2
Related research article	For a published article: S. H. Kim, W. Jung, D. Lim and Y. H. Park, Fault Diagnosis of Ball Bearing Using Dynamic Convolutional Neural Networks Under Varying Speed Condition, in Proceedings of the 48^th^ Annual Conference of the IEEE Industrial Electronics Society, DOI:10.1109/IECON49645.2022.9969003, 2022.

**Value of the Data**

• This article consists of two parts: varying load conditions, and varying speed conditions. In part 1, this dataset contains data related to most of the major faults (bearing, shaft, and rotor faults) that can occur in rotating machines. Therefore, this dataset can be used to verify the performance of the newly developed rotating machine fault diagnosis methods based on rotor dynamics theories.
• In particular, by securing the dataset according to various load conditions, it is possible to observe the change in the fault features according to the load variation. This provides a practical dataset to consider the load fluctuation conditions in the actual field.
• Recently, many fault diagnosis researches using non-contact sensors instead of vibration sensors are being conducted due to the problem of sensor installation and cost in the actual field [1,2]. In this context, this dataset is expected to lead the state-of-the-art fault diagnosis research that utilizes sensor fusion, such as vibration-acoustic or vibration-current.
• In part 2, this dataset was acquired from rolling element bearing under varying speed conditions (680 RPM to 2460 RPM). Three different types of faults, including inner race fault, outer race fault, and ball fault, were seeded. This data consists of vibration data (in the x- and y-directions of the bearing), and current data.
• ·Most of the fault diagnosis methods are proposed for extracting fault features with steady speed and cannot be directly used with varying speed conditions [3]. Practically, in wind turbines, bearing does not operate at a steady speed due to load fluctuations [4]. To solve these problems, eliminating the effect of varying speed condition such as order tracking are conducted, however, it needs to collect synchronized speed data with vibration [5].
• This dataset can be used to develop a learning-based fault diagnosis methodology despite varying speed conditions [6]. Synchronized speed data and vibration data under constant speed conditions are also provided [2].

## 1 Objective

In part 1, this dataset was established for deep learning based rotating machine fault diagnosis research. Unlike other researches, it is very difficult to obtain data in the fault diagnosis research field because it is difficult to apply an actual failure to make training of deep learning algorithms challenging. To solve this problem, we simulated bearing faults, unbalance faults, and misalignment faults that may occurred dominantly in rotating machine. We collected vibration, acoustic, temperature and driving current data under different load conditions (0 Nm, 2 Nm, and 4 Nm). This dataset is measured based on mechanical engineering knowledge in accordance with ISO international standards. This dataset can be used for the verification of newly-developed learning-based fault diagnosis methods.

In part 2, this dataset was established for learning-based ball bearing fault diagnosis research. Unlike other researches with constant speed, it is very difficult to obtain data under the varying speed condition. In contrast, we obtained faulty vibration and driving current data under varying speed conditions (680 RPM to 2460 RPM). This dataset is measured based on mechanical engineering knowledge in accordance with ISO international standards. This dataset can be used for verification of the learning-based fault diagnosis method.

## 2 Data Description

This article presents two varying operating condition including varying load condition and varying speed condition. First dataset consists of vibration, acoustic, temperature and driving current data under varying load conditions. Vibration, temperature, motor current, and acoustic data are collected under 3 different load conditions (0 Nm, 2 Nm and 4 Nm). The load conditions are controlled by hysteresis brake with air cooling method. The main motor rotates at a rated rotating speed of 3010 RPM.

Vibration data were measured using four accelerometers (PCB352C34) at two bearing housings (A, B) in the x-direction and y-direction, simultaneously. An acoustic microphone (PCB378B02) was located nearby the bearing housing (A). Temperature and driving current data were measured using two thermocouples (K-type) and three CT sensors (Hioki CT6700). Siemens SCADAS Mobile 5PM50 was used for collecting vibration and acoustic data. NI9211, and NI9775 modules were used for collecting temperature, and driving current data, respectively. Vibration, temperature, driving current data were collected at a sampling frequency of 25.6 kHz. This dataset was collected for 120 seconds in normal state, and for 60 seconds in faulty state. Lastly, acoustic data were collected with a sampling frequency of 51.2 kHz and only acquire bearing fault data under no-load conditions to avoid the noise from air-cooled brakes.

The collected vibration and acoustic data are stored in binary MATLAB (MAT) files [7,8]. The vibration data file contains five columns, namely ‘Time Stamp’, ‘x_direction_housing_A’, ‘y_direction_housing_A’, ‘x_direction_housing_B’, and ‘y_direction_housing_B’. The unit of the vibration data is ‘gravitational constant (g)’ (1g = 9.80665 m/s^2^). The acoustic data file contains two columns, namely ‘Time Stamp’, and ‘values’. The unit of the acoustic data is ‘Pascal (Pa)’. The description of the vibration and acoustic files as per operating and health conditions of the rotating machine is as follows:

**<**vibration>

(1) *0Nm_Normal.mat: This file includes the vibration data in the x and y directions of two housings of healthy bearing under the 0 Nm load condition.*
(2) *0Nm_BPFI_03.mat: This file includes the vibration data in the x and y directions of two housings of bearing, which has a 0.3 mm inner race fault under the 0 Nm load condition.*
(3) *0Nm_BPFI_10.mat: This file includes the vibration data in the x and y directions of two housings of bearing, which has a 1.0 mm inner race fault under the 0 Nm load condition.*
(4) *0Nm_BPFI_30.mat: This file includes the vibration data in the x and y directions of two housings of bearing, which has a 3.0 mm inner race fault under the 0 Nm load condition.*
(5) *0Nm_BPFO_03.mat: This file includes the vibration data in the x and y directions of two housings of bearing, which has a 0.3 mm outer race fault under the 0 Nm load condition.*
(6) *0Nm_BPFO_10.mat: This file includes the vibration data in the x and y directions of two housings of bearing, which has a 1.0 mm outer race fault under the 0 Nm load condition.*
(7) *0Nm_BPFO_30.mat: This file includes the vibration data in the x and y directions of two housings of bearing, which has a 3.0 mm outer race fault under the 0 Nm load condition.*
(8) *0Nm_Misalign_01.mat: This file includes the vibration data in the x and y directions of two housings of bearing, which has a 0.1 mm misalignment fault under the 0 Nm load condition.*
(9) *0Nm_Misalign_03.mat: This file includes the vibration data in the x and y directions of two housings of bearing, which has a 0.3 mm misalignment fault under the 0 Nm load condition.*
(10) *0Nm_Misalign_05.mat: This file includes the vibration data in the x and y directions of two housings of bearing, which has a 0.5 mm misalignment fault under the 0 Nm load condition.*
(11) *0Nm_Unbalance_0583mg.mat: This file includes the vibration data in the x and y directions of two housings of bearing, which has a 583 mg unbalance fault under the 0 Nm load condition.*
(12) *0Nm_Unbalance_1169mg.mat: This file includes the vibration data in the x and y directions of two housings of bearing, which has a 1169 mg unbalance fault under the 0 Nm load condition.*
(13) *0Nm_Unbalance_1751mg.mat: This file includes the vibration data in the x and y directions of two housings of bearing, which has a 1751 mg unbalance fault under the 0 Nm load condition.*
(14) *0Nm_Unbalance_2239mg.mat: This file includes the vibration data in the x and y directions of two housings of bearing, which has a 2239 mg unbalance fault under the 0 Nm load condition.*
(15) *0Nm_Unbalance_3318mg.mat: This file includes the vibration data in the x and y directions of two housings of bearing, which has a 3318 mg unbalance fault under the 0 Nm load condition.*
(16) *2Nm_Normal.mat: This file includes the vibration data in the x and y directions of two housings of healthy bearing under the 2 Nm load condition.*
(17) *2Nm_BPFI_03.mat: This file includes the vibration data in the x and y directions of two housings of bearing, which has a 0.3 mm inner race fault under the 2 Nm load condition.*
(18) *2Nm_BPFI_10.mat: This file includes the vibration data in the x and y directions of two housings of bearing, which has a 1.0 mm inner race fault under the 2 Nm load condition.*
(19) *2Nm_BPFI_30.mat: This file includes the vibration data in the x and y directions of two housings of bearing, which has a 3.0 mm inner race fault under the 2 Nm load condition.*
(20) *2Nm_BPFO_03.mat: This file includes the vibration data in the x and y directions of two housings of bearing, which has a 0.3 mm outer race fault under the 2 Nm load condition.*
(21) *2Nm_BPFO_10.mat: This file includes the vibration data in the x and y directions of two housings of bearing, which has a 1.0 mm outer race fault under the 2 Nm load condition.*
(22) *2Nm_BPFO_30.mat: This file includes the vibration data in the x and y directions of two housings of bearing, which has a 3.0 mm outer race fault under the 2 Nm load condition.*
(23) *2Nm_Misalign_01.mat: This file includes the vibration data in the x and y directions of two housings of bearing, which has a 0.1 mm misalignment fault under the 2 Nm load condition.*
(24) *2Nm_Misalign_03.mat: This file includes the vibration data in the x and y directions of two housings of bearing, which has a 0.3 mm misalignment fault under the 2 Nm load condition.*
(25) *2Nm_Misalign_05.mat: This file includes the vibration data in the x and y directions of two housings of bearing, which has a 0.5 mm misalignment fault under the 2 Nm load condition.*
(26) *2Nm_Unbalance_0583mg.mat: This file includes the vibration data in the x and y directions of two housings of bearing, which has a 583 mg unbalance fault under the 2 Nm load condition.*
(27) *2Nm_Unbalance_1169mg.mat: This file includes the vibration data in the x and y directions of two housings of bearing, which has a 1169 mg unbalance fault under the 2 Nm load condition.*
(28) *2Nm_Unbalance_1751mg.mat: This file includes the vibration data in the x and y directions of two housings of bearing, which has a 1751 mg unbalance fault under the 2 Nm load condition.*
(29) *2Nm_Unbalance_2239mg.mat: This file includes the vibration data in the x and y directions of two housings of bearing, which has a 2239 mg unbalance fault under the 2 Nm load condition.*
(30) *2Nm_Unbalance_3318mg.mat: This file includes the vibration data in the x and y directions of two housings of bearing, which has a 3318 mg unbalance fault under the 2 Nm load condition.*
(31) *4Nm_Normal.mat: This file includes the vibration data in the x and y directions of two housings of healthy bearing under the 4 Nm load condition.*
(32) *4Nm_BPFI_03.mat: This file includes the vibration data in the x and y directions of two housings of bearing, which has a 0.3 mm inner race fault under the 4 Nm load condition.*
(33) *4Nm_BPFI_10.mat: This file includes the vibration data in the x and y directions of two housings of bearing, which has a 1.0 mm inner race fault under the 4 Nm load condition.*
(34) *4Nm_BPFI_30.mat: This file includes the vibration data in the x and y directions of two housings of bearing, which has a 3.0 mm inner race fault under the 4 Nm load condition.*
(35) *4Nm_BPFO_03.mat: This file includes the vibration data in the x and y directions of two housings of bearing, which has a 0.3 mm outer race fault under the 4 Nm load condition.*
(36) *4Nm_BPFO_10.mat: This file includes the vibration data in the x and y directions of two housings of bearing, which has a 1.0 mm outer race fault under the 4 Nm load condition.*
(37) *4Nm_BPFO_30.mat: This file includes the vibration data in the x and y directions of two housings of bearing, which has a 3.0 mm outer race fault under the 4 Nm load condition.*
(38) *4Nm_Misalign_01.mat: This file includes the vibration data in the x and y directions of two housings of bearing, which has a 0.1 mm misalignment fault under the 4 Nm load condition.*
(39) *4Nm_Misalign_03.mat: This file includes the vibration data in the x and y directions of two housings of bearing, which has a 0.3 mm misalignment fault under the 4 Nm load condition.*
(40) *4Nm_Misalign_05.mat: This file includes the vibration data in the x and y directions of two housings of bearing, which has a 0.5 mm misalignment fault under the 4 Nm load condition.*
(41) *4Nm_Unbalance_0583mg.mat: This file includes the vibration data in the x and y directions of two housings of bearing, which has a 583 mg unbalance fault under the 4 Nm load condition.*
(42) *4Nm_Unbalance_1169mg.mat: This file includes the vibration data in the x and y directions of two housings of bearing, which has a 1169 mg unbalance fault under the 4 Nm load condition.*
(43) *4Nm_Unbalance_1751mg.mat: This file includes the vibration data in the x and y directions of two housings of bearing, which has a 1751 mg unbalance fault under the 4 Nm load condition.*
(44) *4Nm_Unbalance_2239mg.mat: This file includes the vibration data in the x and y directions of two housings of bearing, which has a 2239 mg unbalance fault under the 4 Nm load condition.*
(45) *4Nm_Unbalance_3318mg.mat: This file includes the vibration data in the x and y directions of two housings of bearing, which has a 3318 mg unbalance fault under the 4 Nm load condition.*

<acoustic>

(1) *0Nm_Normal.mat: This file includes the acoustic data of healthy bearing under the 0 Nm load condition.*
(2) *0Nm_BPFI_03.mat: This file includes the acoustic data of bearing which has a 0.3 mm inner race fault under the 0 Nm load condition.*
(3) *0Nm_BPFI_10.mat: This file includes the acoustic data of bearing which has a 1.0 mm inner race fault under the 0 Nm load condition.*
(4) *0Nm_BPFO_03.mat: This file includes the acoustic data of bearing which has a 0.3 mm outer race fault under the 0 Nm load condition.*
(5) *0Nm_BPFO_10.mat: This file includes the acoustic data of bearing which has a 1.0 mm outer race fault under the 0 Nm load condition.*

The collected temperature and driving current data are stored in technical data management streaming (TDMS) files [9,10]. Temperature and driving current data of each condition are stored in the one data file. Temperature and motor current data file contains six columns namely ‘Time Stamp’, ‘Temperature_housing_A’, ‘Temperature_housing_B’, ‘U-phase’, ‘V-phase’, and ‘W-phase’. The units of the temperature and motor current are ‘Celsius (°C), and ‘ampere (A)’, respectively. The description of the temperature and motor current files as per operating and health conditions of the rotating machine is as follows:

<current, temperature>

(1) *0Nm_Normal.tdms: This file includes the temperature data in two housings and the current data of three phases of healthy bearing under the 0 Nm load condition.*
(2) *0Nm_BPFI_03.tdms: This file includes the temperature data in two housings and the current data of three phases of bearing, which has a 0.3 mm inner race fault under the 0 Nm load condition.*
(3) *0Nm_BPFI_10.tdms: This file includes the temperature data in two housings and the current data of three phases of bearing, which has a 1.0 mm inner race fault under the 0 Nm load condition.*
(4) *0Nm_BPFI_30.tdms: This file includes the temperature data in two housings and the current data of three phases of bearing, which has a 3.0 mm inner race fault under the 0 Nm load condition.*
(5) *0Nm_BPFO_03.tdms: This file includes the temperature data in two housings and the current data of three phases of bearing, which has a 0.3 mm outer race fault under the 0 Nm load condition.*
(6) *0Nm_BPFO_10.tdms: This file includes the temperature data in two housings and the current data of three phases of bearing, which has a 1.0 mm outer race fault under the 0 Nm load condition.*
(7) *0Nm_BPFO_30.tdms: This file includes the temperature data in two housings and the current data of three phases of bearing, which has a 3.0 mm outer race fault under the 0 Nm load condition.*
(8) *0Nm_Misalign_01.tdms: This file includes the temperature data in two housings and the current data of three phases of bearing, which has a 0.1 mm misalignment fault under the 0 Nm load condition.*
(9) *0Nm_Misalign_03.tdms: This file includes the temperature data in two housings and the current data of three phases of bearing, which has a 0.3 mm misalignment fault under the 0 Nm load condition.*
(10) *0Nm_Misalign_05.tdms: This file includes the temperature data in two housings and the current data of three phases of bearing, which has a 0.5 mm misalignment fault under the 0 Nm load condition.*
(11) *0Nm_Unbalance_0583mg.tdms: This file includes the temperature data in two housings and the current data of three phases of bearing, which has a 583 mg unbalance fault under the 0 Nm load condition.*
(12) *0Nm_Unbalance_1169mg.tdms: This file includes the temperature data in two housings and the current data of three phases of bearing, which has a 1169 mg unbalance fault under the 0 Nm load condition.*
(13) *0Nm_Unbalance_1751mg.tdms: This file includes the temperature data in two housings and the current data of three phases of bearing, which has a 1751 mg unbalance fault under the 0 Nm load condition.*
(14) *0Nm_Unbalance_2239mg.tdms: This file includes the temperature data in two housings and the current data of three phases of bearing, which has a 2239 mg unbalance fault under the 0 Nm load condition.*
(15) *0Nm_Unbalance_3318mg.tdms: This file includes the temperature data in two housings and the current data of three phases of bearing, which has a 3318 mg unbalance fault under the 0 Nm load condition.*
(16) *2Nm_Normal.tdms: This file includes the temperature data in two housings and the current data of three phases of healthy bearing under the 2 Nm load condition.*
(17) *2Nm_BPFI_03.tdms: This file includes the temperature data in two housings and the current data of three phases of bearing, which has a 0.3 mm inner race fault under the 2 Nm load condition.*
(18) *2Nm_BPFI_10.tdms: This file includes the temperature data in two housings and the current data of three phases of bearing, which has a 1.0 mm inner race fault under the 2 Nm load condition.*
(19) *2Nm_BPFI_30.tdms: This file includes the temperature data in two housings and the current data of three phases of bearing, which has a 3.0 mm inner race fault under the 2 Nm load condition.*
(20) *2Nm_BPFO_03.tdms: This file includes the temperature data in two housings and the current data of three phases of bearing, which has a 0.3 mm outer race fault under the 2 Nm load condition.*
(21) *2Nm_BPFO_10.tdms: This file includes the temperature data in two housings and the current data of three phases of bearing, which has a 1.0 mm outer race fault under the 2 Nm load condition.*
(22) *2Nm_BPFO_30.tdms: This file includes the temperature data in two housings and the current data of three phases of bearing, which has a 3.0 mm outer race fault under the 2 Nm load condition.*
(23) *2Nm_Misalign_01.tdms: This file includes the temperature data in two housings and the current data of three phases of bearing, which has a 0.1 mm misalignment fault under the 2 Nm load condition.*
(24) *2Nm_Misalign_03.tdms: This file includes the temperature data in two housings and the current data of three phases of bearing, which has a 0.3 mm misalignment fault under the 2 Nm load condition.*
(25) *2Nm_Misalign_05.tdms: This file includes the temperature data in two housings and the current data of three phases of bearing, which has a 0.5 mm misalignment fault under the 2 Nm load condition.*
(26) *2Nm_Unbalance_0583mg.tdms: This file includes the temperature data in two housings and the current data of three phases of bearing, which has a 583 mg unbalance fault under the 2 Nm load condition.*
(27) *2Nm_Unbalance_1169mg.tdms: This file includes the temperature data in two housings and the current data of three phases of bearing, which has a 1169 mg unbalance fault under the 2 Nm load condition.*
(28) *2Nm_Unbalance_1751mg.tdms: This file includes the temperature data in two housings and the current data of three phases of bearing, which has a 1751 mg unbalance fault under the 2 Nm load condition.*
(29) *2Nm_Unbalance_2239mg.tdms: This file includes the temperature data in two housings and the current data of three phases of bearing, which has a 2239 mg unbalance fault under the 2 Nm load condition.*
(30) *2Nm_Unbalance_3318mg.tdms: This file includes the temperature data in two housings and the current data of three phases of bearing, which has a 3318 mg unbalance fault under the 2 Nm load condition.*
(31) *4Nm_Normal.tdms: This file includes the temperature data in two housings and the current data of three phases of healthy bearing under the 4 Nm load condition.*
(32) *4Nm_BPFI_03.tdms: This file includes the temperature data in two housings and the current data of three phases of bearing, which has a 0.3 mm inner race fault under the 4 Nm load condition.*
(33) *4Nm_BPFI_10.tdms: This file includes the temperature data in two housings and the current data of three phases of bearing, which has a 1.0 mm inner race fault under the 4 Nm load condition.*
(34) *4Nm_BPFI_30.tdms: This file includes the temperature data in two housings and the current data of three phases of bearing, which has a 3.0 mm inner race fault under the 4 Nm load condition.*
(35) *4Nm_BPFO_03.tdms: This file includes the temperature data in two housings and the current data of three phases of bearing, which has a 0.3 mm outer race fault under the 4 Nm load condition.*
(36) *4Nm_BPFO_10.tdms: This file includes the temperature data in two housings and the current data of three phases of bearing, which has a 1.0 mm outer race fault under the 4 Nm load condition.*
(37) *4Nm_BPFO_30.tdms: This file includes the temperature data in two housings and the current data of three phases of bearing, which has a 3.0 mm outer race fault under the 4 Nm load condition.*
(38) *4Nm_Misalign_01.tdms: This file includes the temperature data in two housings and the current data of three phases of bearing, which has a 0.1 mm misalignment fault under the 4 Nm load condition.*
(39) *4Nm_Misalign_03.tdms: This file includes the temperature data in two housings and the current data of three phases of bearing, which has a 0.3 mm misalignment fault under the 4 Nm load condition.*
(40) *4Nm_Misalign_05.tdms: This file includes the temperature data in two housings and the current data of three phases of bearing, which has a 0.5 mm misalignment fault under the 4 Nm load condition.*
(41) *4Nm_Unbalance_0583mg.tdms: This file includes the temperature data in two housings and the current data of three phases of bearing, which has a 583 mg unbalance fault under the 4 Nm load condition.*
(42) *4Nm_Unbalance_1169mg.tdms: This file includes the temperature data in two housings and the current data of three phases of bearing, which has a 1169 mg unbalance fault under the 4 Nm load condition.*
(43) *4Nm_Unbalance_1751mg.tdms: This file includes the temperature data in two housings and the current data of three phases of bearing, which has a 1751 mg unbalance fault under the 4 Nm load condition.*
(44) *4Nm_Unbalance_2239mg.tdms: This file includes the temperature data in two housings and the current data of three phases of bearing, which has a 2239 mg unbalance fault under the 4 Nm load condition.*
(45) *4Nm_Unbalance_3318mg.tdms: This file includes the temperature data in two housings and the current data of three phases of bearing, which has a 3318 mg unbalance fault under the 4 Nm load condition.*

Second, the collected dataset consists of vibration and current data acquired from the ball bearing with different fault types of inner race faults, outer race faults, and ball faults, according to changes in motor speed conditions (680 RPM and 2460 RPM).

Vibration data were measured using four accelerometers (PCB352C34) at the two bearing housing A and B in the x-direction and y-direction. Current data were measured using three CT current sensors (Hioki CT6700). Vibration data were acquired by a Siemens SCADAS Mobile 5PM50 with sampling frequency of 25.6 kHz, and current data were acquired by NI9775 with sampling frequency of 100 kHz. This dataset was collected for 600 seconds at constant speed, and for 2,100 seconds at varying speed conditions (680 RPM and 2460 RPM).

**Fig. 1:**
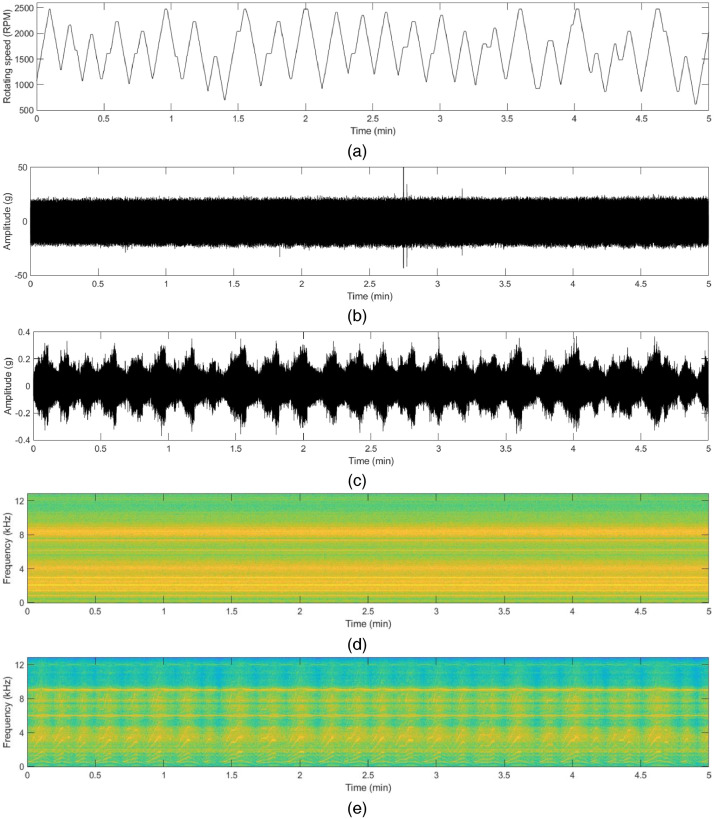
Vibration (acceleration) data in x and y-direction in normal state: (a) rotating speed of the motor, (b) time-series acceleration data with constant rotating speed, (c) time-series acceleration data with varying rotating speed, (d) corresponding spectrogram with the constant rotating speed, (e) corresponding spectrogram with varying rotating speed.

**Fig. 2:**
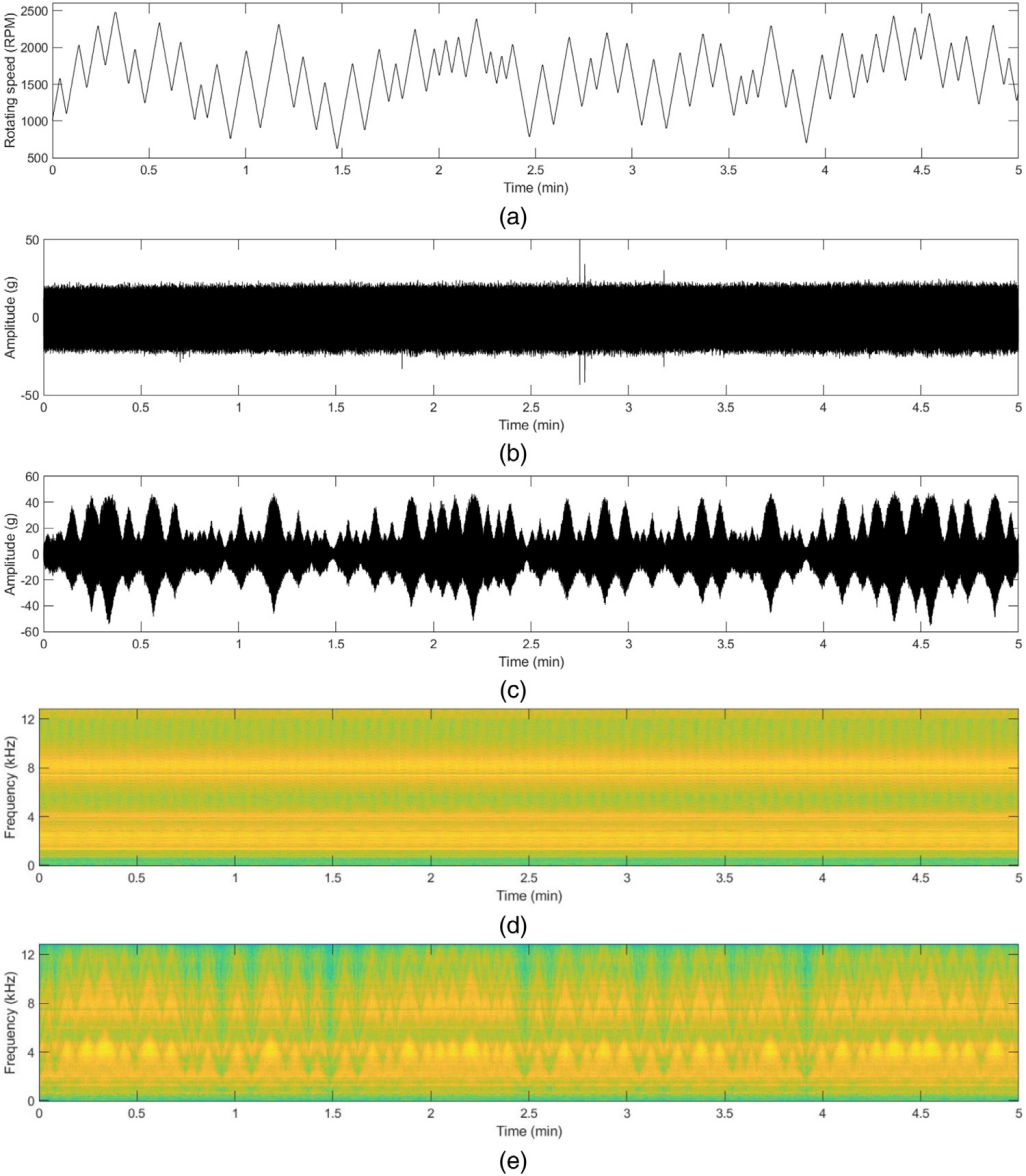
Vibration (acceleration) data in x and y-direction in inner race fault state: (a) rotating speed of the motor, (b) time-series acceleration data with constant rotating speed, (c) time-series acceleration data with varying rotating speed, (d) corresponding spectrogram with the constant rotating speed, (e) corresponding spectrogram with varying rotating speed.

**Fig. 3:**
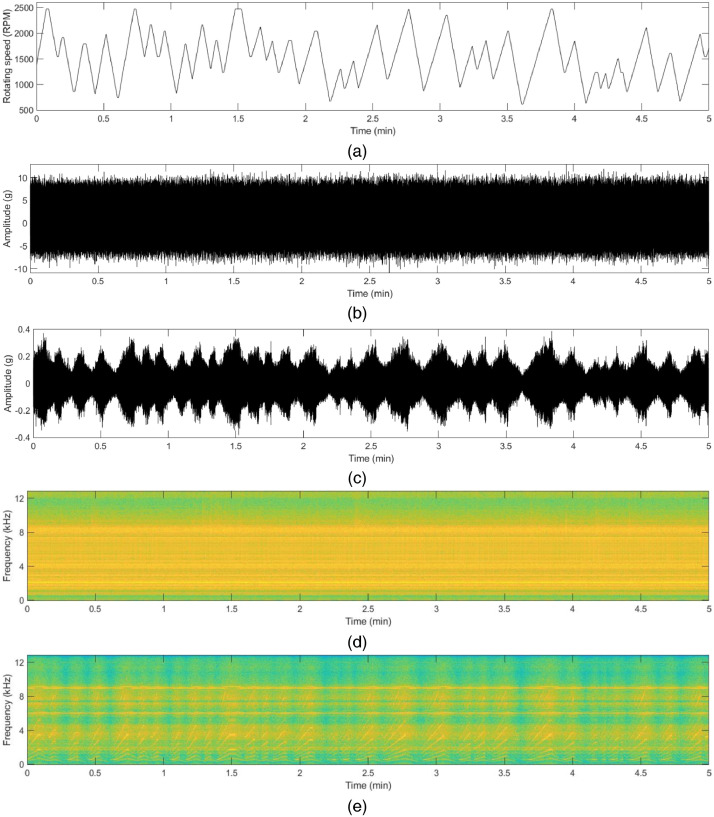
Vibration (acceleration) data in x and y-direction in outer race fault state: (a) rotating speed of the motor, (b) time-series acceleration data with constant rotating speed, (c) time-series acceleration data with varying rotating speed, (d) corresponding spectrogram with the constant rotating speed, (e) corresponding spectrogram with varying rotating speed.

**Fig. 4:**
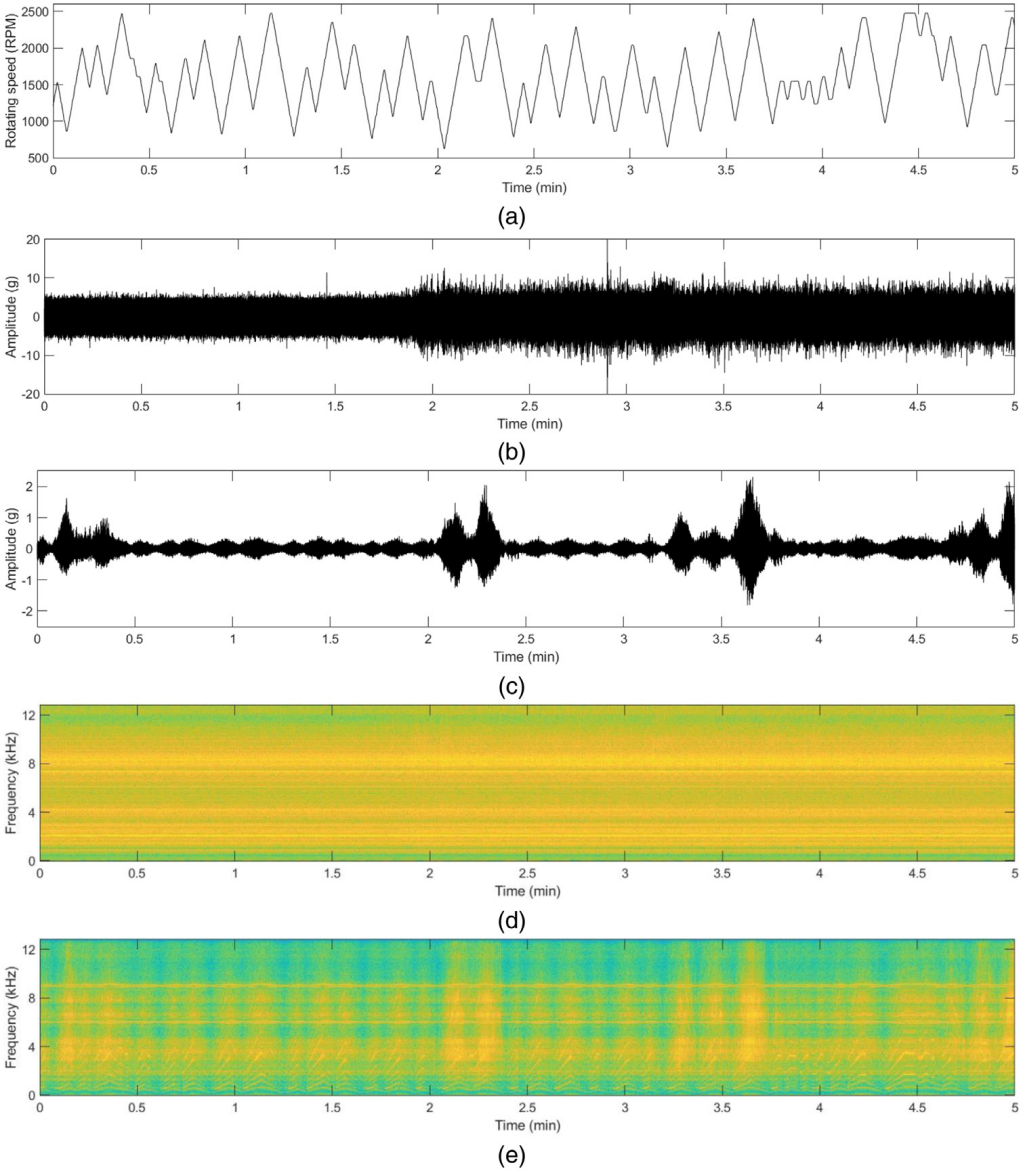
Vibration (acceleration) data in x and y-direction in ball fault state: (a) rotating speed of the motor, (b) time-series acceleration data with constant rotating speed, (c) time-series acceleration data with varying rotating speed, (d) corresponding spectrogram with the constant rotating speed, (e) corresponding spectrogram with varying rotating speed.

The vibration data file contains five columns, namely ‘Time Stamp’, ‘x_direction_housing_A’, ‘y_direction_housing_A’, ‘x_direction_housing_B’, and ‘y_direction_housing_B’. The unit of the vibration data is ‘gravitational constant (g)’ (1g = 9.80665 m/s^2^). The motor current data file contains five columns, namely ‘Time Stamp’, ‘R_phase’, ‘S_phase’ and ‘T_phase’. The unit of the motor current is ‘Ampere (A)’. To support more details in this dataset, synchronized speed data are also provided. Sample raw data and their time-frequency analysis of each state are shown in Figs. 1 to 4. The description of the dataset is as follows:

*<vibration>*

(46) *vibration_normal_constant.csv: This file includes the 600 seconds length of vibration data of the x and y directions of two bearing housings of normal under the constant speed condition at 3010 RPM.*
(47) *vibration_inner/outer/ball_constant.csv: This file includes the 600 seconds length of vibration data of the x and y directions of two bearing housings of inner/outer/ball fault under the constant speed condition at 3010 RPM. Bearing with ball fault is installed in bearing housing A.*
(48) *vibration_normal_0.csv ∼ vibration_normal_6.csv: Each file includes the 300 seconds length of vibration data of the x and y directions of two bearing housings under the varying speed condition.*
(49) *vibration_inner_0.csv ∼ vibration_inner_6.csv: Each file includes the 300 seconds length of vibration data of the x and y directions of two bearing housings under the varying speed condition. Bearing with inner fault is installed in bearing housing B.*
(50) *vibration_outer_0.csv ∼ vibration_outer_6.csv: Each file includes the 300 seconds length of vibration data of the x and y directions of two bearing housings under the varying speed condition. Bearing with outer fault is installed in bearing housing B.*
(51) *vibration_ball_0.csv ∼ vibration_ball_6.csv: Each file includes the 300 seconds length of vibration data of the x and y directions of two bearing housings under the varying speed condition. Bearing with ball fault is installed in bearing housing B.*

***<*** *Motor current>*

(1) *current_normal_0.csv ∼ current_normal_6.csv: Each file includes the 300 seconds length of current data of the R-, S-, and T-phase of main motor under the varying speed condition.*
(2) *current_inner_0.csv ∼ current_inner_6.csv: Each file includes the 300 seconds length of current data of the R-, S-, and T-phase of main motor under the varying speed condition. Bearing with inner fault is installed in bearing housing B.*
(3) *current_outer_0.csv ∼ current_outer_6.csv: Each file includes the 300 seconds length of current data of the R-, S-, and T-phase of main motor under the varying speed condition. Bearing with outer fault is installed in bearing housing B.*
(4) *current_ball_0.csv ∼ current_ball_6.csv: Each file includes the 300 seconds length of current data of the R-, S-, and T-phase of main motor under the varying speed condition. Bearing with ball fault is installed in bearing housing B.*

*<rotating speed (RPM)>*

(1) *rpm_normal_0.csv ∼ rpm_normal_6.csv: Each file includes the 300 seconds length of rotating speed of main motor under the varying speed condition.*
(2) *rpm_inner_0.csv ∼ rpm_inner_6.csv: Each file includes the 300 seconds length of rotating speed of main motor under the varying speed condition. Bearing with inner fault is installed in bearing housing B.*
(3) *rpm_outer_0.csv ∼ rpm_outer_6.csv: Each file includes the 300 seconds length of rotating speed of main motor under the varying speed condition. Bearing with outer fault is installed in bearing housing B.*
(4) *rpm_ball_0.csv ∼ rpm_ball_6.csv: Each file includes the 300 seconds length of rotating speed of main motor under the varying speed condition. Bearing with ball fault is installed in bearing housing B.*

## 3 Experimental Design, Materials and Methods

### 3.1 Section 1: Description of Testbed

**Fig. 5:**
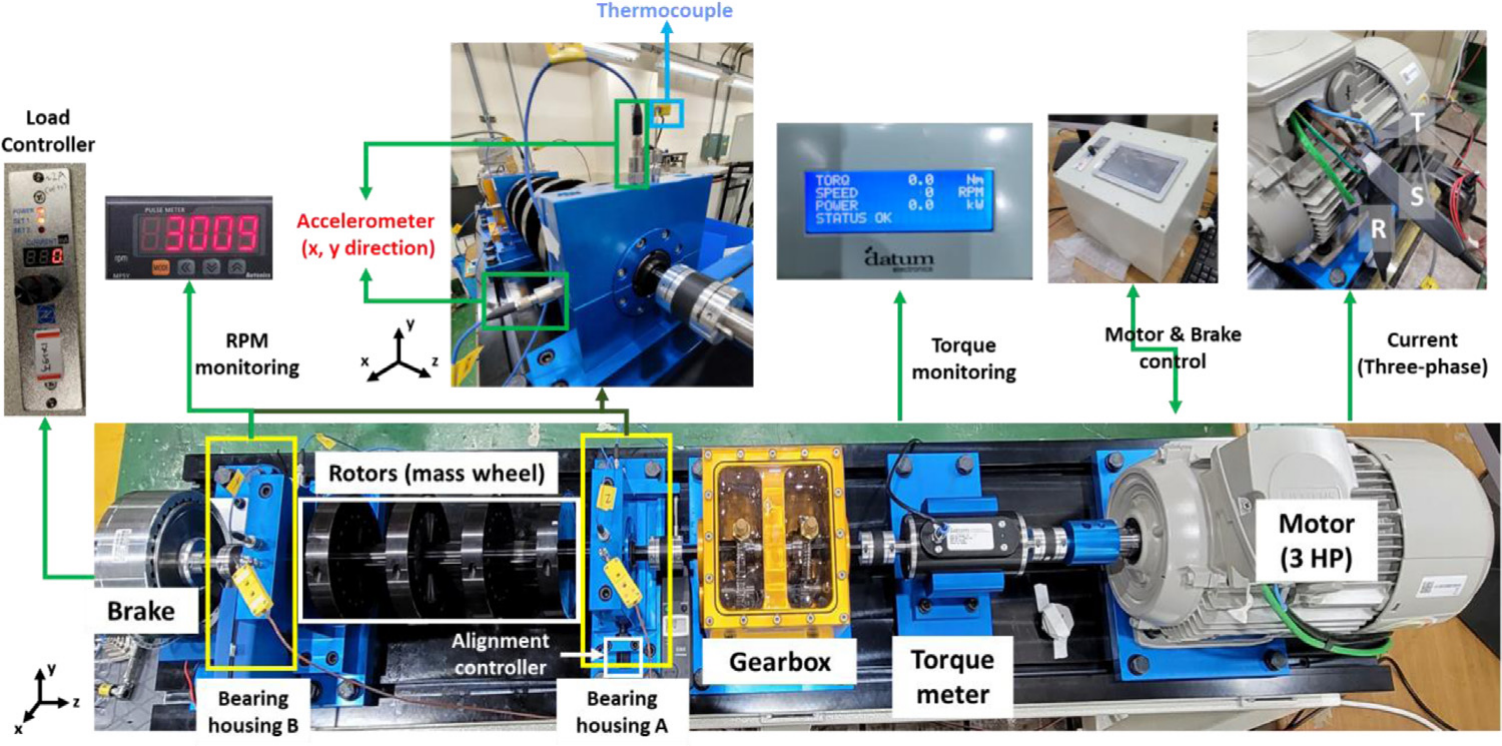
Layout of the rotating machine testbed and its components.

The rotating machine testbed consists of three-phase induction motor, torque meter, gearbox, bearing housing A, bearing housing B, rotors and hysteresis brake as shown in Fig. 5. The three-phase induction motor manufactured by SIEMENS is four-pole AC motor with 3 horse-power (HP). It is driven at 380 V, 60 Hz, at a rated speed of 1770 rpm. The gearbox increases the rotating speed by 2.07 times, up to 3663 rpm. To avoid signal overlap with the driving frequency of 60 Hz, this dataset was operated at 3010 rpm. A load was applied to the rotating machine using a hysteresis brake (AHB-3A) manufactured by Valid Magnetic Ltd., and the load was measured with a torque meter (M425) manufactured by Datum Electronics. The simulated loads in this dataset are 0 Nm, 2 Nm, and 4 Nm. Acoustic data were collected under zero-load conditions, as the brake is an air-cooling method and can act as noise to the microphone.

A total of four accelerometers (PCB35234) were installed in the x- and y-directions of bearing housings A and B, based on the vibration installation guide (ISO 10816-1:1995). A microphone (PCB378B02) was located nearby bearing housing A based on the microphone installation guide (ISO 8528-10). Two thermocouples (K-type) were installed in each bearing housing to measure the bearing temperature. To measure the three-phase motor current, three CT sensors (Hioki CT6700) were used. CT sensors were installed on the U-phase, V-phase, and W-phase of the three-phase motor.

### 3.2 Section 2: Method of Fault Seedings

Rolling element bearings are composed of two concentric rings called races and rolling elements such as balls or rollers between the races. The inner and outer raceway are grooved. To assemble a ball bearing, the balls are inserted in between the inner race and the outer race. The inner race is snapped to a position concentric with the outer race. The balls are separated uniformly between the races, and a riveted cage is inserted to maintain the separation.

**Fig. 6:**
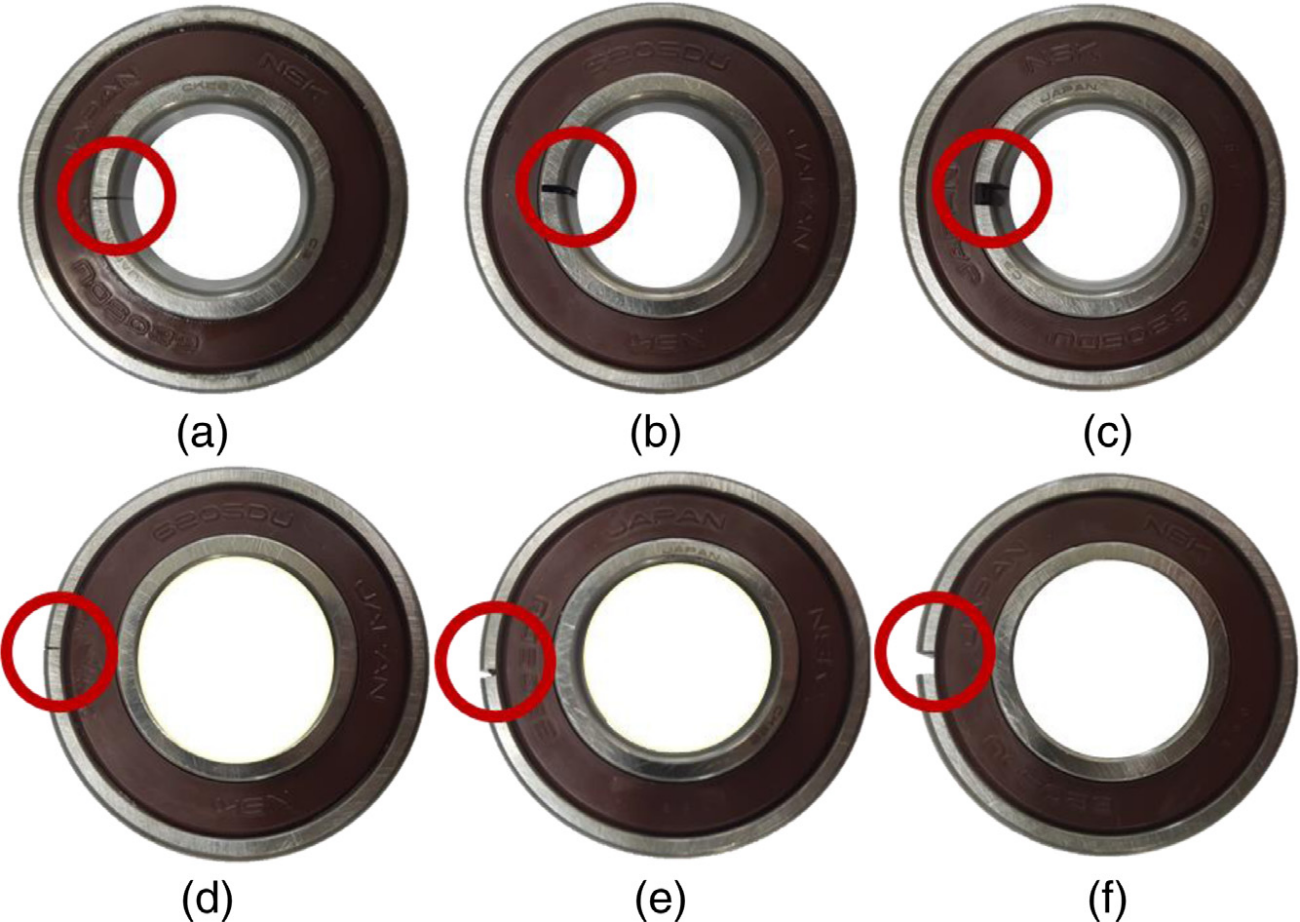
Bearing by crack size: (a) inner race 0.3 mm, (b) inner race 1.0 mm, (c) inner race 3.0 mm, (d) outer race 0.3 mm, (e) outer race 1.0 mm, and (f) outer race 3.0 mm

In varying load condition test, the bearing faults, including inner race fault and outer race fault were simulated according to the crack sizes (0.3 mm, 1.0 mm, and 3.0 mm) as shown in Fig. 6. The corresponding fault-seeded bearing was installed in the bearing housing A. Depending on the rotating speed, the bearing faults frequencies can be calculated as described in Table 1 [11]. This dataset uses standardized NSK bearing (NSK 6205 DDU) with a ball diameter (*d*) of 7.90 mm, a pitch diameter (*D*) of 38.5 mm, contact degree angle (*θ*) of zero degrees, and the number of balls (*N*) is 9. Therefore, the shaft frequency (*f_s_*) is 50.17 Hz, fundamental train frequency (FTF) is 19.94 Hz, ball pass frequency inner (BPFI) is 272.07 Hz, ball pass frequency outer (BPFO) is 179.43 Hz, and ball spin frequency (BSF) is 234.19 Hz.

**Table 1:** Characteristic fault frequencies for ball bearing.

Characteristic frequency	Equation
Fundamental train frequency (FTF)	$\frac{f_{s}}{2}\left(1-\frac{d}{D}\cos\theta \right)$
Ball pass frequency inner (BPFI)	$\frac{Nf_{s}}{2}\left(1+\frac{d}{D}\cos\theta \right)$
Ball pass frequency outer (BPFO)	$\frac{Nf_{s}}{2}\left(1-\frac{d}{D}\cos\theta \right)$
Ball spin frequency (BSF)	$\frac{Df_{s}}{d}\left[1-{\left(\frac{d}{D}\right)}^{2}\cos\theta \right]$

**Fig. 7:**
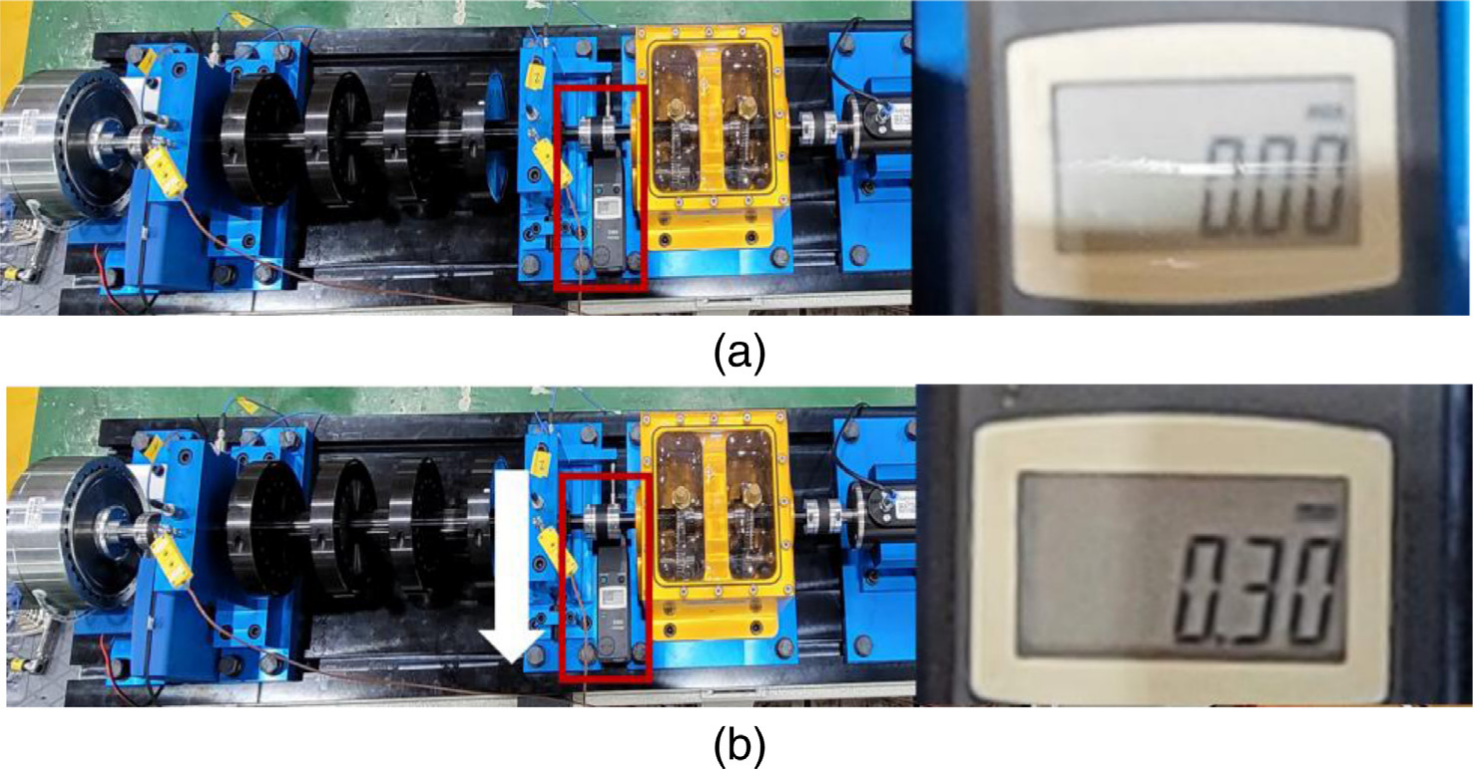
Description of shaft misalignment: (a) normal, and (b) shaft misalignment that 0.3 mm misaligned in direction of the white arrow.

**Fig. 8:**
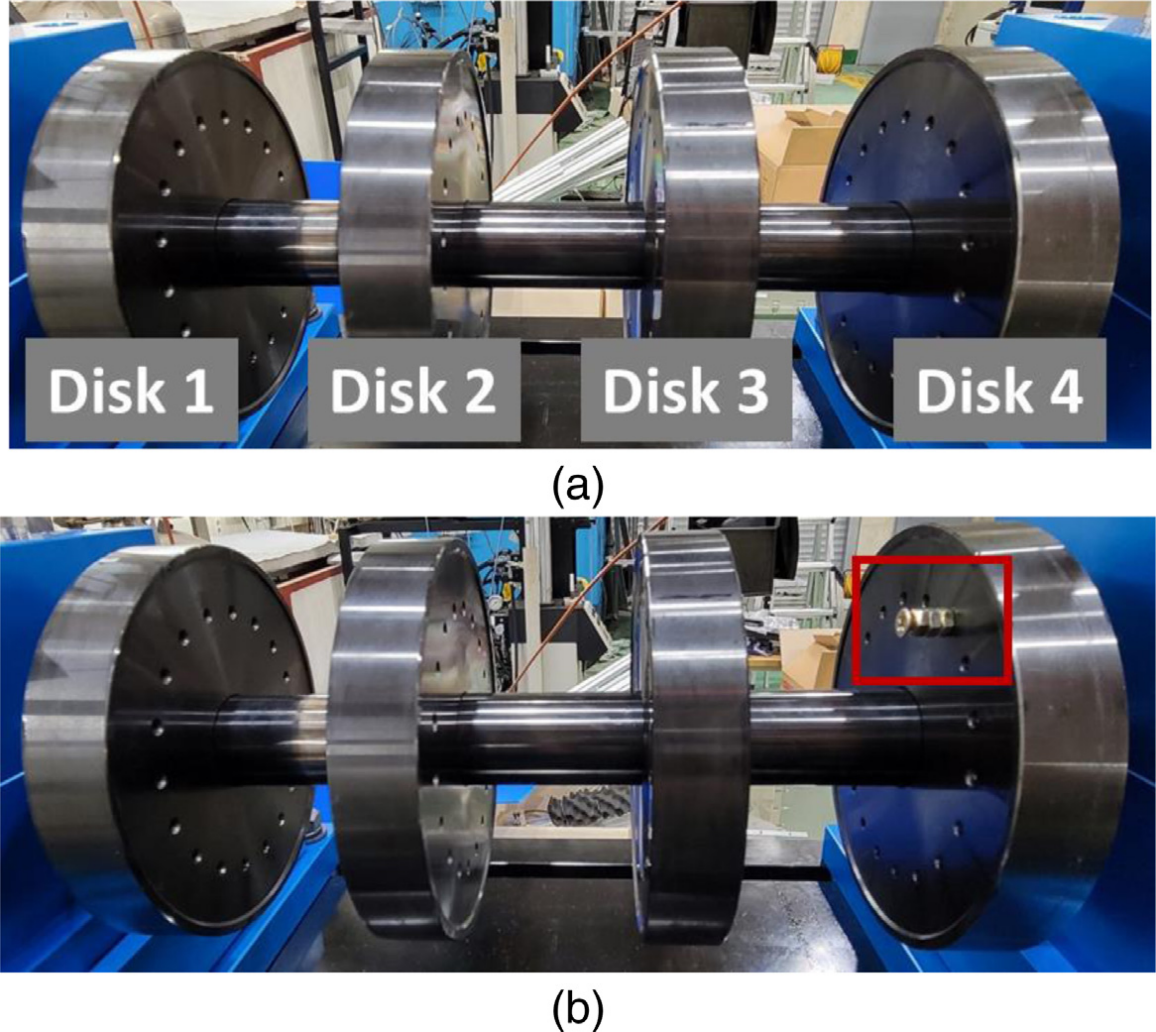
Description of rotor unbalance: (a) normal, and (b) rotor unbalance on the fourth disk.

Shaft fault is a parallel misalignment that moves the shaft in bearing housing A as shown in Fig. 7. The movements consist of 0.1 mm, 0.3 mm, and 0.5 mm. Rotor faults are seeded by adding mass to the fourth rotor disk for simulating mass unbalance as shown in Fig. 8. The unbalanced disk is the closest disk to bearing housing A. This dataset consists of the unbalanced masses: 583 milligram (mg), 1169 mg, 1751 mg, 2239 mg, and 3318 mg. The overall description of dataset is listed in Table 2.

**Table 2:** Description of dataset where ‘Inner’ is bearing with inner race fault, and ‘Outer’ is bearing with outer race fault.

Data types	Fault location	Fault types	Fault severity	Length (second)	Load (Nm)	Rotating speed (RPM)	Sampling rate (kHz)
Vibration, Temperature, Motor current	-	Normal	-	120	0, 2, 4	3010	25.6
	Disk 4	Unbalance	583 mg	60	0, 2, 4	3010	25.6
			1169 mg	60	0, 2, 4	3010	25.6
			1751 mg	60	0, 2, 4	3010	25.6
			2239 mg	60	0, 2, 4	3010	25.6
			3318 mg	60	0, 2, 4	3010	25.6
	Bearing housing A	Misalignment	0.1 mm	60	0, 2, 4	3010	25.6
			0.3 mm	60	0, 2, 4	3010	25.6
			0.5 mm	60	0, 2, 4	3010	25.6
		Inner	0.3 mm	60	0, 2, 4	3010	25.6
			1.0 mm	60	0, 2, 4	3010	25.6
			3.0 mm	60	0, 2, 4	3010	25.6
		Outer	0.3 mm	60	0, 2, 4	3010	25.6
			1.0 mm	60	0, 2, 4	3010	25.6
			3.0 mm	60	0, 2, 4	3010	25.6

Acoustic	-	Normal	-	60	0	3010	51.2
	Bearing housing A	Inner	0.3 mm	60	0	3010	51.2
			1.0 mm	60	0	3010	51.2
		Outer	0.3 mm	60	0	3010	51.2
			1.0 mm	60	0	3010	51.2

**Fig. 9:**
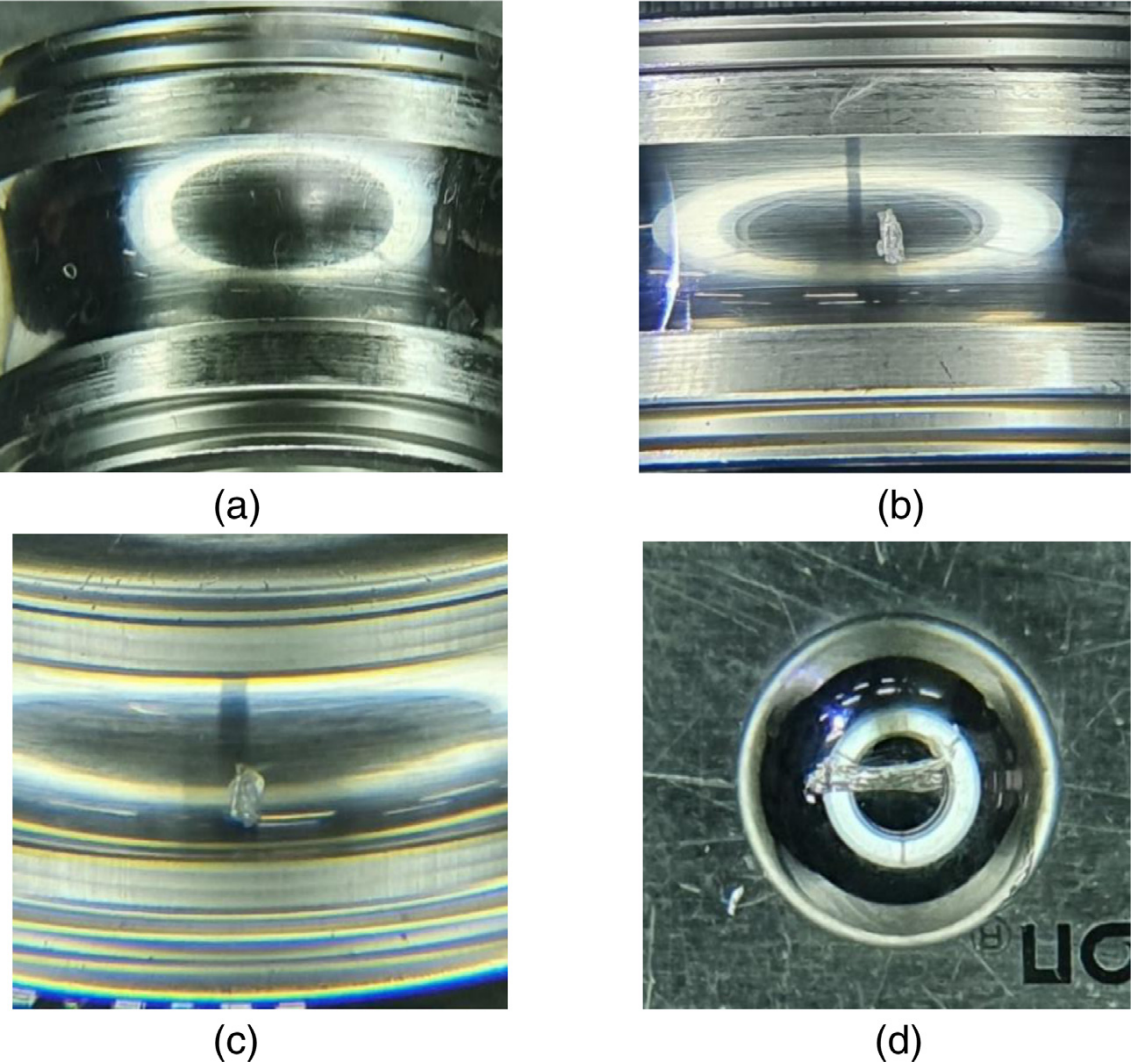
The condition of bearing: (a) normal, (b) inner race fault, (c) outer race fault and (d) ball fault.

In varying speed condition test, type 6205 steel NSK ball bearing were used for testing. Four different state (normal, inner race faults, outer race faults and ball faults) of the ball bearing were emulated as shown in Fig. 9. These faults were generated by spalling surface of ball bearing using diamond tips. The varying speed conditions was simulated by adjusting the frequency of the motor as shown in Fig. 5. The overall description of dataset is shown in Table 3.

**Table 3:** Description of dataset where ‘Inner’ is bearing with inner race fault, ‘Outer’ is bearing with outer race fault, and ‘Ball’ is bearing with ball fault.

Data types	Speed condition	Fault location	Fault types	Length (second)	Load (Nm)	Rotating speed (RPM)	Sampling rate (kHz)
Vibration	Constant	Bearing housing A	Normal	600	0	3010	25.6
			Inner	600	0	3010	25.6
			Outer	600	0	3010	25.6
			Ball	600	0	3010	25.6
	Varying	Bearing housing B	Normal	2,100 (300 per file)	0	680 ∼ 2460	25.6
			Inner	2,100 (300 per file)	0	680 ∼ 2460	25.6
			Outer	2,100 (300 per file)	0	680 ∼ 2460	25.6
			Ball	2,100 (300 per file)	0	680 ∼ 2460	25.6

Motor current	Varying	Bearing housing B	Normal	2,100 (300 per file)	0	680 ∼ 2460	100
			Inner	2,100 (300 per file)	0	680 ∼ 2460	100
			Outer	2,100 (300 per file)	0	680 ∼ 2460	100
			Ball	2,100 (300 per file)	0	680 ∼ 2460	100

## Ethics Statements

Human Lab., Center for Noise and Vibration Control Plus, Department of Mechanical Engineering, Korea Advanced Institute of Science and Technology, Daejeon, South Korea has given the consent that the datasets may be publicly-released as part of this publication.

## CRediT authorship contribution statement

**Wonho Jung:** Conceptualization, Methodology, Software, Validation, Visualization, Writing – original draft. **Seong-Hu Kim:** Data curation, Validation, Investigation. **Sung-Hyun Yun:** Data curation, Validation, Investigation. **Jaewoong Bae:** Data curation, Validation, Investigation. **Yong-Hwa Park:** Funding acquisition, Writing – review & editing, Supervision.

## Declaration of Competing Interest

The authors declare that they have no known competing financial interests or personal relationships that could have appeared to influence the work reported in this paper.

## Data Availability

Vibration, Acoustic, Temperature, and Motor Current Dataset of Rotating Machine Under Varying Load Conditions for Fault Diagnosis (Original data) (Mendeley Data). Vibration and Motor Current Dataset of Rolling Element Bearing Under Varying Speed Conditions for Fault Diagnosis: Subset1 (Original data) (Mendeley Data). Vibration and Motor Current Dataset of Rolling Element Bearing Under Varying Speed Conditions for Fault Diagnosis: Subset2 (Original data) (Mendeley Data). Vibration and Motor Current Dataset of Rolling Element Bearing Under Varying Speed Conditions for Fault Diagnosis: Subset3 (Original data) (Mendeley Data).
